# Impact of oil prices, the U.S interest rates on Turkey’s real estate market. New evidence from combined co-integration and bootstrap ARDL tests

**DOI:** 10.1371/journal.pone.0242672

**Published:** 2021-01-04

**Authors:** Ahmed Alhodiry, Husam Rjoub, Ahmed Samour

**Affiliations:** 1 Banking and Finance Department, Cyprus International University, Nicosia, North Cyprus; 2 Banking and Finance Department, Near East University, Nicosia, North Cyprus; University of Almeria, SPAIN

## Abstract

The research aims to provide new empirical evidence by testing the impact of the external shocks namely: oil prices and the U.S interest rate on Turkey’s real estate market by using three techniques of co-integration tests namely: the newly developed bootstrap autoregressive distributed lag (ARDL) testing approach as proposed by (McNown et al. 2018), the new approach involving the Bayer-Hanck (2013) combined co-integration test, Hatemi-J (2008) co-integration testing approach. The ARDL model is utilized to explore the relationship between the variables. The findings show that the oil prices have a positive impact on Turkey’s real estate market, the results confirm that there is a significant impact of oil prices on Turkey’s real estate market through the domestic interest rate. Furthermore, the results demonstrated that there is a significant spillover influence of the U.S. interest rates on Turkey’s real estate market through oil prices and domestic interest rates. This study suggests that the following factors led to increasing the sensitivity and volatility of the Turkish real estate market to oil prices and the U.S. interest rate fluctuations: the presence of economic interdependence between the USA and Turkey, and the majority of the external debts and the reserve currency in Turkey are composed in the USD, and Turkey’s oil imports hit record high in last years. Finally, this article suggests that policymakers in Turkey should pay close attention to the effects of external shocks namely the oil prices and U.S. interest rates on Turkish markets to maintain economic and financial stability.

## Introduction

Over the past decades, Turkey’s integration into the world markets has increased, resulting in the globalization of the world economy. The Turkish markets are easily affected by the policies of developed economies or any global external shocks such as oil prices. In this regard, the main objective of this research is to provide new empirical evidence by testing the impact of the external shocks namely: The U. S interest rate and oil prices on Turkey’s real estate market.

Turkey has limited oil reserves, and the oil production in Turkey was reported at 314,000 Barrel/Day in 1980 and reached to 731,000 Barrel/Day in 2014. However, Turkey’s demand for crude oil from global markets has increased constantly in the last 30 years. In 2016, Turkey’s total liquid fuel consumption averaged about 861,000 Barrel/Day and more than 90% of total crude oil came from imports. 70% of Turkey’s imports of crude oil came from Iraq, Iran, and Russia [1]. However, Turkey has faced several challenges in energy security, the first main challenge is the energy supply problem. Turkey’s main energy suppliers are Russia and Iran. Probable any economic or political disagreements with these countries put energy security in Turkey at risk [2]. In this regard, Turkey should try to find new suppliers of oil sources to diversify the suppliers to reduce the dependency on the main suppliers. The second main challenge is the high dependency on imported oil, domestic oil production in Turkey is not enough to meet the country’s energy needs. Despite the limitation of oil production, the oil demand rapidly increasing. The rate of imported oil must be decreased by finding more renewable energy sources for the energy supply formula. At this point, Turkey should evaluate its alternatives to renewable energy sources such as solar, wind, and geothermal. However, these resources can simply be produced and renewed. Also, it diffuses fewer pollutants to nature, and it can never be depleted worldwide. Renewable energy resources in Turkey are hydroelectric, wind solar, geothermal, biomass, and waves [3]. Turkey is the third country in the world with 1.28 million tons of oil equivalent (MTOE) in terms of producing geothermal energy worldwide, especially the Aegean territory has huge geothermal energy potential [2].

Although the advantages of renewable energy resources. Non-renewable consumption of energy in Turkey has consistently elevated with the increase in its population. Nonrenewable energy consumption (kg of oil equivalent) has increased around 220% over the period from 1980 to 2014. Energy consumption was 715,149 kg of oil equivalent in 1980 and reached 1,577,828 kg of oil equivalent in 2014. However, the economy of this country is one of the leading producers in the world in textiles, ships, motor vehicles, consumer electronics, home appliances, construction materials, and other transportation equipment, thus led to increasing energy consumption namely oil consumption. According to the literature, the variations of oil prices have a significant impact on various economic variables. In this regard: [4–7] have indicated that the effect of the oil prices on economic indexes in developed and developing countries can be different; these different findings can be attributed to various economic factors for instance: (oil-importing countries vs oil-exporting countries). Increases in oil prices can be economically bad for oil-importing countries but it is economically good news for oil-exporting countries. However, Turkey is a country that imports a large part of its oil needs from abroad. Hence an increase in oil prices negatively affects Turkey’s current account balance and economic growth and other economic variables [8]. In this regard, if crude oil prices go up, then inflation in oil-importing countries like Turkey goes up, which leads to an increase in interest rates. Thus, any increase in interest rate can affect the cost of finance, which in turn leads to an increase in the housing prices. In this sense, the main objective of this research is to provide new empirical evidence by testing the impact of the crude oil prices on Turkey’s real estate market through the domestic interest rate channel. Besides, the study aims to test the impact of the U.S interest rate on Turkey’s real estate market through the oil prices and the domestic interest rate.

The shocks from developed economies like the U.S. economy can affect Turkey’s economy through various transmission mechanisms, such as interest rate and exchange rate channels. The Turkish markets have the potential for the volatility of financial markets due to any change in the U.S. monetary policy, particularly the interest rates channel. Because any increase in the U.S. interest rate, investors will continue to withdraw their investment of emerging markets like Turkey. Therefore, any change in U.S. interest rates may affect the Turkish markets [9]. This article suggests that the influence of the U.S. interest rates on Turkey’s real estate market depends on how this rate affect capital outflows, international trade, exchange rate, and any excess volatility of exchange rate, financial flows, and international trade can negatively affect the economic and financial stability, particularly on Turkey’s real estate market. However, this article aims to analyze the influence of international spillover effects of the U.S. interest rates and the influence of domestic interest rates on Turkey’s real estate market.

In the last decades, Turkey has faced several critical reforms, such as ensuring the operational independence of the central bank to support the banking sector and the financial market and eliminating any restrictions on the capital inflow. As a result of these reforms, international trade as a share of Gross Domestic Product (GDP) has increased from 18% in 1981 to 61% in 2018 and the total exports have risen from 2.9 billion USD in 1981 to 166.5 billion USD in 2018. Furthermore, housing assets in Turkey have experienced spectacular growth over the last decades. The housing market represents the largest asset category for Turkish households, with a 70% housing ratio in 2018 [2]. However, real estate market developments stand out as one of the considerable economic concerns in Turkey, and the vitality in the housing market is considered as the most important indicator of macroeconomic performance. Numerically, the Turkish real market economy offers great investment potential with its value of 19.5% of the total GDP [10]. Besides, the real estate and construction sector occupied 4.1 billion USD and 24.8% of the total FDI. According to the Knight Frank Global House Price Index, Turkey was recorded as the 55 in terms of annual price growth index. Also, urban renewal projects have started in different Turkish cities. It seems approximately 6.7 million residential units are expected to be demolished and rebuilt over the next two decades [10]. Therefore, this situation increases the importance of researches on the variables affecting the real estate market. The purpose of this article is to provide empirical evidence of the effects of oil prices on Turkey’s real estate market. Furthermore, this study aims to test the impact of the U.S. interest rates on emerging markets like Turkey, particularly after the 2008–2009 global financial crisis (GFC).

Many empirical studies have explored the importance of the real estate market in Turkey. Some of these studies have examined the linkage between the real estate market and macroeconomic activity. This relation has drawn special attention in the literature as housing investment has been considered as a substantial leading indicator of economic activity, especially after the 2008 GFC. The GFC in 2008 has increased concerns about economic stability in Turkey and puts the spotlight on the linkage between financial markets and macroeconomic variables (exchange rate, interest rate) and the spillover influence of the external factors (the U.S. interest rates).

After the 2008–2009 crisis, the central bank of Turkey started to monitor financial markets development more closely and has started some macro-prudential measures into monetary policy channels to address and overcome these concerns. Therefore, the credit provided by the banking sectors to the markets as a percentage of GDP increased approximately 70% between 2010 and 2018, and the stock of housing credits as a percentage of GDP ratio increased over the period from 2010 to 2018 and reached above 10% by the end of 2018. The increases in housing credits are expected to continue in the future.

In the literature, the impact of changes in interest rate channels on housing prices is defined as the housing price channel of monetary policy [11]. In this regard, the monetary policy can affect the real estate market through various channels (direct and indirect mechanisms). In the direct mechanisms, the monetary policy channels can affect the real estate market through the user cost of housing, housing supply, and expectations of future housing prices movement. However, the user cost of housing effect is considered as the main direct impact of monetary policy on the real estate market. On this basis, when the interest rate increases, the average mortgage rate also increases. Thus, an increase in the average mortgage rate leads to an increase in the user cost of capital and an increase in the user cost will lead to a decrease in demand size for housing, leading to a fall in housing prices in the market. Furthermore, an increase in interest rate may have a significant effect on housing construction costs, causing a decline in the housing output [12].

In indirect mechanisms, the monetary policy channels can affect the real estate market through standard credit-channel [13]. On this basis, when the interest rate is increased, the individual housing wealth value falls as real housing prices decline due to a decrease in demand for houses. A decline in individuals’ wealth may lead to a decline in housing demand, leading to lower housing prices. Contrarily, an increase in interest rates will lead to an increase in mortgage repayments, leading to a decline in credit-constrained households’ cash flow and eventually to a decrease in housing prices [14].

The structure of this research is designed as follows: section (2) of this article shows an overview of the literature review; sections (3 and 4) are data, methodology, and findings of the results; and section (5) is the main conclusion of this the article.

### Contributions to the current literature

The research aims to provide new empirical evidence by testing the impact of the external shocks namely: oil prices and the U.S interest rate on Turkey’s real estate market by using three techniques of co-integration tests namely: the newly developed bootstrap autoregressive distributed lag (ARDL) testing approach as proposed by (McNown et al. 2018), the new approach involving the Bayer-Hanck (2013) combined co-integration test, Hatemi-J (2008) co-integration testing approach. The ARDL model is utilized to explore the relationship between the variables. However, our study provides three main contributions to the current literature. First, to the best of our knowledge, no empirical research tested the impact of U.S interest rate on Turkey’s real estate market. Second, the study provides robustness and comprehensive analysis by testing the external shocks namely: oil prices and the U.S interest rate on Turkey’s real estate market by using three techniques of co-integration tests. Third, the study uses a the newly developed bootstrap autoregressive distributed lag (ARDL) testing approach as proposed by (McNown et al. 2018) to test the relationship between the selected variables.

### Literature review

In this article, we focus on two sections. First, the article aims to test the impact of oil prices on the real estate market. According to the literature, the variations of oil prices have a significant influence on various economic variables [4–7].

[4] showed that oil prices have a significant impact on GDP in 12 European countries. [5] tested the impact of oil prices on economic activities in Thailand. The results found that oil prices have a significant effect on macroeconomic variables, such as investment, over the period from 1993 to 2006. [6] examined the impact of oil prices on real income in Turkey. The results showed that any change in oil prices has a powerful impact on real income in Turkey, over the period from 1996–2017. [7] indicated that oil prices have a powerful impact on the exchange rates in Saudi Arabia. Furthermore, several papers have tested the impact of oil prices on the equity market. In this regard, [15] have tested the linkage between oil prices and the equity market in Australia, the study found that there is a significant and positive linkage between oil prices and the equity market in Australia. [16] tested the influence of the oil prices on the equity market of the oil-importing countries. The findings showed that oil price shocks have a powerful impact on the equity market of oil-importing countries. [8] used structural VAR and confirmed a positive linkage between oil prices and s the U.S stock market.

While there are limited empirical papers that have tested the effect of oil prices on real estate markets, [17] tested the effect of oil prices on real estate in Saudi Arabia. By employing Markov switching, the results fount that there is a significant influence of crude oil prices on the real estate market in Saudi Arabia during the period 2008 to 2015. [18] has examined the effect of oil prices on the real estate market in Malaysia. Using the Toda-Yamamoto, the results suggested that oil prices are one of the leading factors responsible for the variation of the Malaysian real estate market. [19] tested the effect of oil inflows on the real estate market in Iran. The findings found that there is a statistical and positive relation between oil inflows and the Iranian real estate market.

Second, the study aims to test the influence of the U.S interest rates on Turkey’s real estate market. The linkage between the interest rates and the real estate market has attracted extensive attention in recent years. However, some empirical studies support a negative link between interest rates and the real estate market [20–24]. In this regard, [20] used data from 1965 to 2005 and tested the impact of the U.S monetary policy channels on the real estate market in the USA. Using the VAR model. The empirical findings indicated that the contractionary monetary policy (increasing the interest rate) harms the U.S. real estate markets. Similarly, [21] used monthly data sets from 2000 to 2010 and investigated the relationships between interest rates and housing prices. The findings indicated a negative linkage between the interest rate channel and the housing market in the US.

[22] used data from 1974Q2 to 2008Q4 and tested the effect of monetary policy channels on the real estate market of Australia. Using the VAR model, the authors found that a contractionary monetary policy significantly reduced the housing activity. The results suggested that investment in the housing sector can be an alternative to investment in stock and bonds, thus leading to an inverse linkage between interest rates and the housing market. [23] tested the effect of interest rate channels on the real estate market in China from 1998 to 2009. The authors showed that lower interest rates had an accelerative impact on house price growth, and suggested that monetary policy tools are the key driving forces behind the changes in house price growth in China. [24] used a standard multivariate dynamic model and tested the relationship between the interest rate and house price in China from 1998 to 2010, and stated that interest rate negatively affects the real estate market in China. The authors suggested that the monetary policy tools in China are the key drivers behind the real estate market fluctuations in China. [25] used the generalized variance decomposition approach and tested the linkage between interest rates and the real estate market in Turkey from 1961 to 2000. The findings shocks of the interest rates had noticeable effects on the real estate market and suggested that housing investment in Turkey is a leading indicator of economic activity. Contrarily, some empirical studies support a positive link between the interest rate and the housing market. On this basis, [26] used a set data from 1987 to 2007 and investigated the effect of the U.S. monetary policy on housing prices in the USA and observed that monetary policy had a positive effect on house prices, and a strong short-lived effect on risk spreads in money and mortgage markets.

[27] tested the causality linkage between interest rate and housing price changes in Malaysia based on the Granger-causality test. The regression findings showed a direction causality relation between the interest rate and house price. [28] has tested the relationship between the interest rate and house prices in Vietnam from 2009 to 2018. Using the ARDL approach to estimate the relation between the interest rate and the housing market, the author showed that the interest rate has a positive influence on the housing market in the short run.

Whereas, some empirical studies showed that there is no link between the interest rate policy and the real estate market. For instance, [29] tested the linkage between the interest rate and the real estate market in New Zealand from 1999 to 2009. Based on the two-stage least squares pool regression, the author showed that an increase in the interest rate policy rate may be ineffective in depression the real estate market.

According to the literature, the influence of the U.S. interest rate on global markets has attracted extensive attention in last years. [30] tested the spillover impact of the U.S. interest rate on financial markets of 12 countries in the Asia-Pacific. The findings showed a significant negative impact of the U.S. interest rate on the financial markets of 12 countries in the Asia-Pacific.

[31] indicated that the U.S. interest rate has a powerful effect on global emerging financial markets. Similar results were found by [32] who indicated that the U.S. interest rate has a powerful influence on many emerging financial economies. Furthermore, this effect has a more powerful impact on markets with economies closely linked to the United States. Similarly, [33] used the VAR model and indicated that there is a significant impact of international spillover influence of the U.S. interest rates on the advanced and emerging economies.

[34] tested the impact of U.S. monetary shocks on interest rates and exchange rates in 26 selected countries. Using the VAR model, the results suggested that countries with more stringent controls experienced smaller currency depreciation. [35] tested the effects of the U.S. interest rates on local interest rates and the exchange rate channels in East Asian countries. Using the VAR estimation techniques, the authors found that the local interest rate channel responds robustly to the U.S. interest rate changes. Contrarily, [36] showed that the U.S. interest rates have no impact on India’s financial markets.

In testing the influence of the U.S. interest rates on the Turkish markets, [9] utilized the ARDL testing model and suggested that the U.S. interest rates have a significant impact on Turkey’s financial markets from 2002 to 2017. These findings indicated that the U.S. interest rates negatively impact the Turkish stock market through debt and interest rate channels. [37] indicated that Turkey’s financial market is significantly correlated with the U.S. financial markets. [38] suggested that U.S. interest rates have a powerful impact on the Turkish banking sector. However, most empirical studies focused on the effect of the U.S. interest rates on the stock market. To the best of our knowledge, this article is the first to test the impact of spillover effects of U.S. interest rates on Turkey’s real estate market using the ARDL testing approach.Table 1 shows the summary of literature review.

**Table 1:** Summary of literature review.

Authors	Methodology	Period	Country	The results
Lardic and Mignon (2006).	Cointegration test	1970–2003	12 European countries	OP increases GDP
Gorus et al. (2017)	Fourier-test	1996–2017	Turkey	OP increases GDP
Mohammed and Abid (2020)	ARDL	1974–2014	Saudi Arabia.	OP affects EX
Faff and Brailsford (1999)	Time seris	1983–1993	Australia.	OP increases SM
Khalfaouia et al. (2019)	GARCH	2010–2010	Selected countries	OP affects SM
Rehman and Serletis (2019)	VAR	2002–2018	USA.	OP affects SM
Alola, (2020)	Markov switching	2008–2015	Saudi Arabia	OP affects SM
Le (2015)	Toda-Yamamoto	1988–2003	Malaysia.	OP affects REM
Khiabani (2015)	VAR	198–2013	Iran	OP affects REM
Silva (2008)	VAR	1965–2005	USA	IR affects REM
McDonald and Stokes (2013)	Time seris	2000–2010	USA	IR affects REM
Wadud et al. (2012)	VAR	1974–2008	Australia	IR affects REM
Xu and Chen (2012)	Time seris	1998–2009	China.	IR affects REM
Sari (2014)	VAR	1961–2001	Turkey	IR affects REM
Tang and Tan (2015)	Time series	2000–2013	Malaysia	IR affects REM
Bui (2020)	ARDL	1998–2018	Vietnam	IR affects REM
Shi et al. (2014)	Time series	1999–2009	New Zealand	IR affects REM
Kim (2009)	Time series	1996–2006	12 selected countries	USI affects SM
Laeven and Tong (2012)	VAR	1990–2008	selected countries	USI affects SM
Samour et al. (2019)	ARDL	2002–2017	Turkey	USI affects SM

OP is oil price, EX is exchange rate, SM is stock market, REM is real estate market, USI is U.S interest rate.

### Methodology

#### Data and model specification

A monthly dataset that spans from August 2009 to August 2018 was employed for this article. The data was retrieved from the organization for economic co-operation and development, and the Central Bank of the Republic of Turkey (TCMB). The main assumption of the research was that the oil prices and domestic interest rates, and the U.S. interest rates affect the real estate market in Turkey. Thus, the main equation of this article can be checked as follows:

$$lnRE_{t}=\;\beta_{0}+\;\beta_{1}\;lnOP_{t}+\;\beta_{2}\;lnDI_{t}+\;\beta_{3}\;lnUSI_{t}+\;\varepsilon_{t}$$ (1)

*lnRE*_*t*_ *and lnDI*_*t*_ represent the logarithm of the real estate market and short-term interest rates in Turkey, *lnOP*_*t*_ is the Brent crude oil price, this is generally utilized in Turkey [1]. the *lnUSI*_*t*_ represents the logarithm of the U.S. interest rates, ε_*t*_ is the error term. However, short-term interest rates have a strong impact on investment opportunities and capital inflow [39].

#### Unit root and co-integration tests

The Dickey and Fuller (1979) [40] unit root test and the Clemente et al. (1998) [41] (CMR) unit root test with (2) structural breaks date (SBD) were utilized to determine the stationary among the examined variables. To examine the effects of oil price, domestic interest rates, and effects of the U.S. interest rates on the Turkey real estate market, the study used autoregressive distributed lag (ARDL). The main advantage of this model is that the ARDL model is more appropriate for small data compared with other cointegration tests [42]. Besides, using the ARDL test, the research aims to demonstrate if the variables are cointegrated in (3) options: at the level I(0), at the first difference I(1) or mixed of I(0), and I(1). The lag length was selected through the Akaike info criterion. In the ARDL model, (F) statistics will be compared to the Pesaran et al. (2001) [42] critical values to capture the cointegration among the examined variables. On this basis, if the value of (F)statistics is higher than the upper bound I(1) the cointegration hypothesis will be accepted. In contrast, the cointegration hypothesis will be rejected if the value of (F)statistics is less than the lower bound I(0). Moreover, if the values of (F)statistics fall between at-level I(0), and the first difference I(1), these values mean that the findings will be indecisive [42].

Recently, this approach upgraded by McNown et al. (2018) [43], the recent version includes additional t-test *t*_*dependent*_ or F-test *F*_*independent*_ on the coefficients of lagged independent variables. The *H*_0_ of *t*_*dependent*_ test is: σ1 = 0. The *H*_1_ of *t*_*dependent*_ test is: *σ*_1_ ≠ 0. While The *H*_0_ of *F*_*independent*_ test is:*H*_0_: *σ*_2_ = *σ*_3_ = *σ*_3_ = *σ*_4_ = *σ*_5_ = 0. The *H*_1_ of *F*_*independent*_ test is:*H*_1_: *σ*_2_ ≠ *σ*_3_ ≠ *σ*_3_ ≠ *σ*_4_ ≠ *σ*_5_ ≠ 0.

The critical values (CV) in the bootstrap ARDL approach, are created based on the specific integration features of each time series data using the procedures of ARDL bootstrap, which in turn lead to eliminating unstable results of the ARDL bounds testing model [43]. However, McNown et al. (2018) upgraded the bootstrap ARDL test by employing a table of CV gained by bootstrap simulation. These steps of the bootstrap test will lead to getting better results than the traditional ARDL bounds test [44]. In particular, the Pesaran et al. (2001) CV allows for (1) variable to be endogenous, while the CV generated with a bootstrap technique allows for the endogeneity of all explanatory examined variables. Also, this approach is more suitable for data includes more than (1) explanatory variable [43].

However, the equation of the ARDL cointegration technique is tested as given below:

$$\begin{array}{l} \Delta lnRE_{t}=\beta_{0}\;+\;\overset{P}{\underset{i=1}{\sum }}\beta_{1}\Delta lnRE_{t}+\overset{q}{\underset{i=1}{\sum }}\beta_{2}\Delta lnOP_{t-i}+\overset{q}{\underset{i=1}{\sum }}\beta_{3}\Delta lnDI_{t-i}+\\ \overset{q}{\underset{i=1}{\sum }}\beta_{4}\Delta lnUSI_{t-i}+\;y_{1}lnRE_{t-1}+y_{2}lnOP_{t-i}+y_{3}lnDI_{t-i}+y_{4}lnUSI_{t-i}\;+e_{t} \end{array}$$ (2)

In Eq (2), lnRE, lnOP, lnDI, and lnUSI are the natural logarithms of examined variables, p represents of number of lags (RE) variable, *q* represents of number of lags (DI and USI) variables; *e*_*t*_ is the error term, and Δ means the operator of the first difference level. The error correction features incorporating long and short-run information in the ARDL model is tested as given below:

$$\begin{array}{l} \Delta lnRE_{t}=\beta_{0}+\;\sum_{i=1}^{n}\beta_{1}\Delta lnRE_{t-i}+\sum_{i=1}^{n}\beta_{2}\Delta lnOP_{t-i}+\sum_{i=1}^{n}\beta_{3}\Delta lnDI_{t-i}+\\ \sum_{i=1}^{n}\beta_{4}\Delta lnUSI_{t-i}+ECT_{t-1\;}+e_{t} \end{array}$$ (3)

The ECT is significant with a *negative*^−^ sign. However, ECT aims to determine the speed of adjustment from the short-term to the long-term levels. To enhance the findings of the ARDL testing result, the article applied the H-J (2008) co-integration technique test proposed by Hatemi-J (2008) [45]. The H-J (2008) allows two SBD and shows the new critical values tests of the co-integration; namely, ADFt, Z_*a*_t, and Z_*t*_t, and it is tested as given below:

$$y_{t}=\alpha_{0}+\;\alpha_{1\text{Dv}1}+\alpha_{1\text{Dv}2}\;+\;\beta_{0\;xt}+\beta_{1}D_{1xt}+\beta_{2}D_{1xt}+\;e_{t}$$ (4)

where Dv1 and Dv2 represent the dummy variables. In this test, the hypothesis of absence co-integration will not be accepted if the calculated values of the ADFt, *z*_*at*_, *z*_*t*_*t* tests higher than the H-J (2008) critical values.

Furthermore, this article uses the new techniques of combined co-integration tests Bayer and Hanck(2013) [46] to boost the findings of the ARDL test. This test combines four various cointegration technique tests; namely, *EG*1987, *JOH*1988, *BO*1994, and *BA*1998*t* as proposed by [47–50] respectively. Besides, this test includes the Fishe*r*^*F*^statistics (FFS) to provide more conclusive results. The BH (2013) test includes functional estimations through disregarding the feature of multiple testing procedures, and it is tested as given below:

$$EG1987t-JOH1988t=-2[IN(P_{EG1987t})+(P_{JOH1988t})\;]$$ (5)

$$EG1987t-JOH1988t-BO1994t-BA1998t\;=-2[IN(P_{EG1987t})+(P_{JO1988t})+\;(P_{BO1994t})+(P_{BA1998t})]$$ (6)

where p is the values of (*EG*1987*t* − *JOH*1988*t* − *BO*1994*t* − *BA*1998*t*)cointegrations tests. To estimate the long-run cointegration, the FFS will be compared with the BH (2013) critical values. The hypothesis of absence long-run combined co-integration will not be accepted if the FFS values exceed the BH (2013) critical values. Moreover, this article used the followıng diagnostics tests (JB normality, the heteroscedasticity, Breusch–Godfrey serial correlation LM, Ramsey). JB normality test was applied to check the normal distribution of the model. The heteroscedasticity and the Breusch–Godfrey serial correlation LM tests were utilized to check the serial-correlation. Besides, the article used the Ramsey, CUSUM, and the CUSUMSQ tests to check the stability of the model.

Furthermore, based on the VECM, the study uses the Granger causality (GC) approach to demonstrate the direction of the causality among *lnRE*, *lnOP*, *lnDI*, *and lnUSI*. The GC approach includes (ECT) to measure the short-run deviations of the time-series data from the long-run equilibrium path. However, the equation of ECM is tested as given below (eqs 7–10):

$$\Delta lnRE_{t}=\beta_{0}\;+\;\overset{p}{\underset{i.=1}{\sum }}\beta_{1}\Delta lnRE_{t-1}+\overset{q}{\underset{i.=1}{\sum }}\beta_{2}\Delta lnOP_{t-1}+\overset{q}{\underset{i.=1}{\sum }}\beta_{3}\Delta lnDI_{t-1}+\overset{q}{\underset{i.=1}{\sum }}\beta_{4}\Delta lnUSI_{t-1}+\partial_{1}\;ECT_{t-1}+e_{t}$$ (7)

$$\Delta lnOP_{t}=\beta_{0}\;+\;\overset{p}{\underset{i.=1}{\sum }}\beta_{1}\Delta lnOP_{t-1}+\overset{q}{\underset{i.=1}{\sum }}\beta_{2}\Delta lnRE_{t-1}+\overset{q}{\underset{i.=1}{\sum }}\beta_{3}\Delta lnDI_{t-1}+\overset{q}{\underset{i.=1}{\sum }}\beta_{4}\Delta lnUSI_{t-1}+\partial_{1}\;ECT_{t-1}+e_{t}$$ (8)

$$\Delta lnDI_{t}=\beta_{0}\;+\;\overset{p}{\underset{i.=1}{\sum }}\beta_{1}\Delta lnDI_{t-1}+\overset{q}{\underset{i.=1}{\sum }}\beta_{2}\Delta lnRE_{t-1}+\overset{q}{\underset{i.=1}{\sum }}\beta_{3}\Delta lnOP_{t-1}+\overset{q}{\underset{i.=1}{\sum }}\beta_{4}\Delta lnUSI_{t-1}+\partial_{1}\;ECT_{t-1}+e_{t}$$ (9)

$$\Delta lnUSI_{t}=\beta_{0}\;+\;\overset{p}{\underset{i.=1}{\sum }}\beta_{1}\Delta lnUSI_{t-1}+\overset{q}{\underset{i.=1}{\sum }}\beta_{2}\Delta lnRE_{t-1}+\overset{q}{\underset{i.=1}{\sum }}\beta_{3}\Delta lnOP_{t-1}+\overset{q}{\underset{i.=1}{\sum }}\beta_{4}\Delta lnDI_{t-1}+\partial_{1}\;ECT_{t-1}+e_{t}$$ (10)

To test the causality relation in the short run: the Wald-testing technique (F.statistics) is used to capture the significance of linked estimated coefficient using the Δ stationary variables. To test the causality relation in the long run: the t-test of the lagged ECT is employed.

#### Empirical findings

Tables 2 and 3 represent the outcomes of the unit root test (ADF and CMR), the outcomes show that the variables of this article (RE, OP, DI, and USI) are integrated at the first level I(1). The findings from Table 4 show the results of the bootstrap ARDL model, the results show that the hypothesis (no cointegration) is rejected, meaning that the cointegration exists between oil price, domestic interest rate, the U.S. interest rate, and Turkey’s real estate market.

**Table 2:** Results of the ADF unit roots test.

I(0)	t -stat,	C.V	I(1)	t-stat,	CV
*lnRE*	-1.921383	-2.890327	*ΔlnRE*	-3.68407**	-2.890327
*lnOP*	-1.352131	-2.21761	*ΔlnOP*	-4.133621**	-2.889753
*lnDI*	-1.469808	-2.889753	*ΔlnDI*	-4.526361**	-2.889753
*lnUSI*	-0.242563	-2.890037	*ΔlnUSI*	-6.703715**	-2.890037

** denotes significance at 5%.

**Table 3:** Results of the CMR test.

	t-stat	C.value	SBD1	SBD2
*lnRE*	-2.729	-5.49	2010M12	2014M01
*lnOP*	-3.113	-5.49	2009M11	2010M08
*lnDI*	-3.345	-5.49	2010M09	2011M06
*lnUSI*	-3.659	-5.49	2012M05	2013M08
*ΔlnRE*	-6.95**	-5.49	2010M11	2015M04
*ΔlOP*	-9.115**	-5.49	2010M10	2011M01
*ΔlDI*	-13.624**	-5.49	2015M11	2016M07
*ΔlUSI*	-6.960**	-5.49	2013M09	2014M11

** denotes significance at 5%.

**Table 4:** The results of bootstrap ARDL approach.

ARDL(4,1,0, 1)		*F*_*pesaran*_	*t*_*dependent*_	*F*_*independent*_
*F lnRE* /*lnOP*, *lnDI*, *lnUIS*,		6.10***	-3.14***	5.62***
Bootstrap-based table CV	1%	3.99	-3.88	7.06
	5%	3.28	-3.08	4.85
	10%	2.94	-2.85	3.96

***,**,* statistical sign at 1%,5%,10% level respectively.

The result of the HJ (2008) cointegration test includes two SBD as shown in Table 5. The result shows that the estimated statistics exceeds the 5% critical value. Therefore, the results provided evidence to reject the null hypothesis (no cointegration) at a 5% significance level.

**Table 5:** Results of HJ (2008) cointegration test with 2 SBD.

	t-stat	SBD1	SBD2	5% Critical values
AD*F*^*test*^	-6.81	2010M9	2010M12	−7.35
Z*T*^*test*^	-11.28**	2013M10	2015M4	−7.35
Z*A*^*test*^	-193.6**	2013M10	2015M4	−104.86

** denotes significance at 5%.

The findings of the BH (2013) test are presented in Table 6. The outcomes indicate that the value of the computed (F)statistics exceeds the calculated (F)statistics in both EG1987T-JO1988T and EG1987T-JO1988T- BO1994T-BA1998T at 5% level of significance. However, the results of BH (2013) and HJ (2008) cointegration tests showed significant evidence to support the ARDL results and confirmed the presence of a long-run association among RE, OP, DI, and USI, and they correspond together in the long-term.

**Table 6:** Results of BH (2013) cointegration test.

	Fisher F statistics		Cointegration
	EG1987T-JO1988T	EG1987T-JO1988T-BO1994T-BA1998T	
	14.954**	21.152**	Cointegration exits
*Sig level at (5%)*	10.72	20.79	

Note:

** denotes significance at 5%.

**Fig 1:**
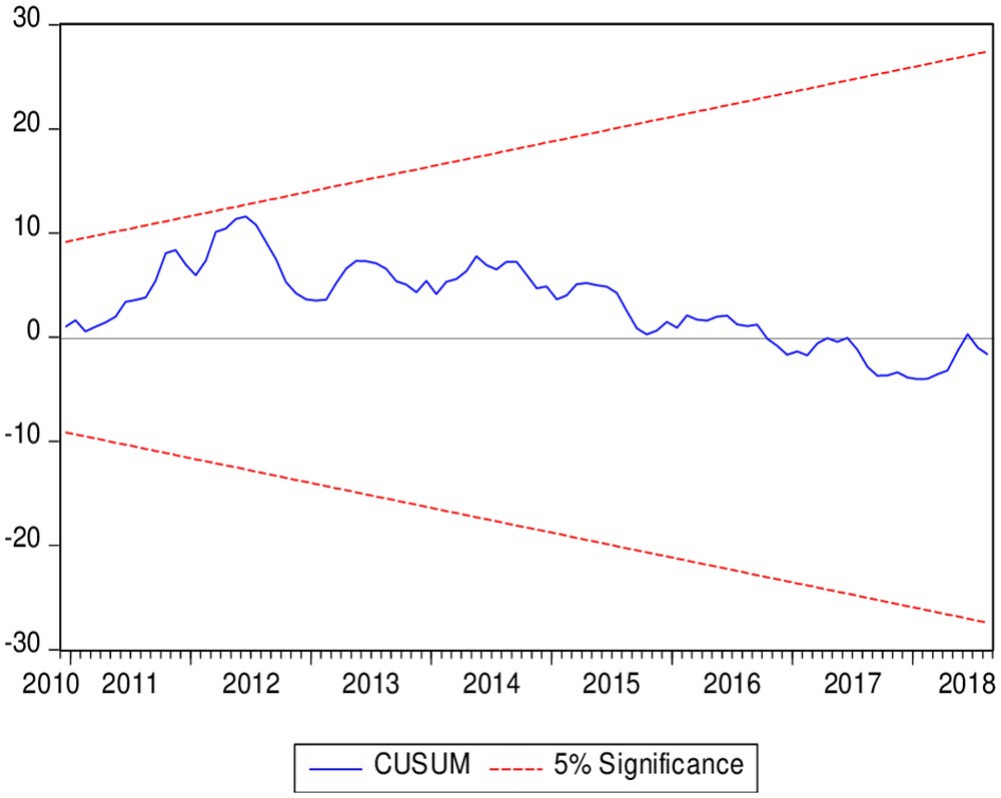
Stability test using (CUSUM).

**Fig 2:**
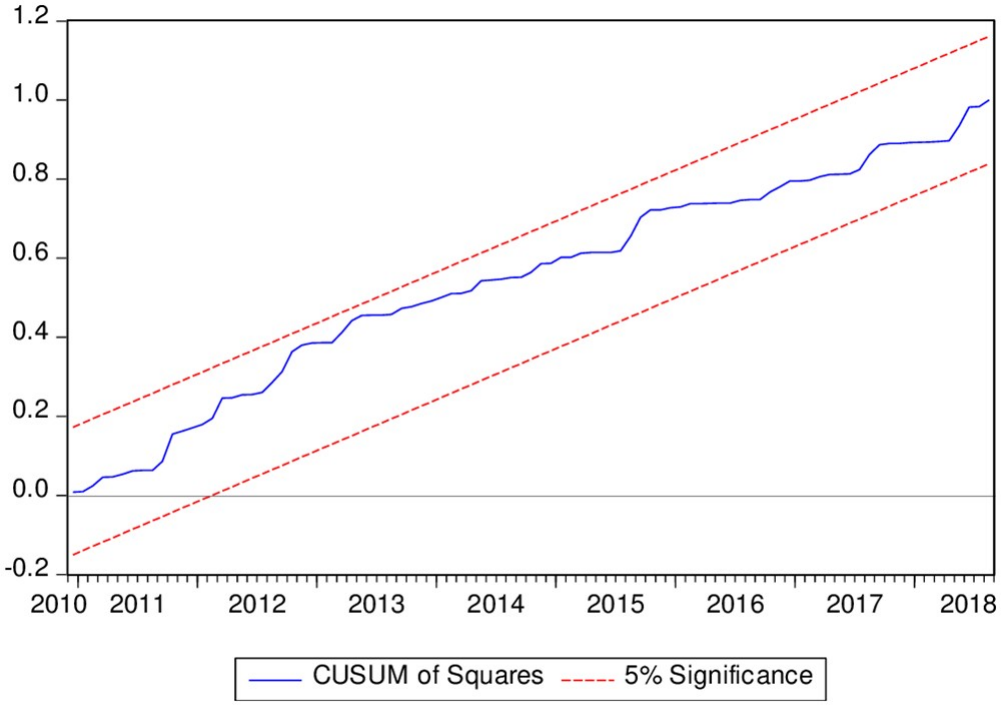
Stability test using (CUSUMQ).

The outcomes of the diagnostic tests are presented in Table 7. The normality test results showed that P-value exceeds the 5% sig level, and it provides evidence that the model of this article is normally distributed. Furthermore, the results of the LM test indicated that there is no autocorrelation in the tested model and this model is homoscedastic. Besides, the Ramsey-Reset test results suggested that the model is well specified. Furthermore, Figs 1 and 2 show the CUSUM and CUSUM of Squares (CUSUMSQ) charts. The CUSUM chart suggests that the model of this article is not miss specified and CUSUM of Squares shows that there is no structural change in the model over the investigated period.

**Table 7:** Results of short and long-term coefficients (ARDL model).

Regressor	Coeff.	t-stat
*ΔlnOP*	0.011**	2.715
*ΔlnDI*	-0.312*	-3.615
*ΔlnUSI*	-0.911**	-1.168
*lnOP*	0.052***	2.615
*lnDI*	-0.002***	-2.115
*lnUSI*	-0.006*	-1.933
ECTt−1	-0.044***	-4.369
*Adjusted R2*	0.969	
*Durbin Watson(-stat*	2.001	
Diagnostics statistics for ARDL model		
JB normality test 0.489(0.716)		
B-Godfrey serial-correlation 0.601(0.311)		
heteroscedasticity (BPG) F-test 1.315(0.182)		
Ramsey RESET test 2.12 (0.140)		

***, **,* denote significance at 1%, 5% and 10%.

The coefficients of the ARDL test are presented in Table 7. The outcomes from short- and long-run estimations indicated a significant and positive effect of the oil prices on Turkey’s real estate market. The results in line with [8] who suggested that there is a significant and positive linkage between oil prices and the real estate market. On the other hand, the results indicated a significant and negative effect of the domestic interest rates on Turkey’s real estate market. The coefficient of interest rates confirms the significant impact of the monetary policy in maintaining economic stability in Turkey using the domestic interest rates channel. This finding is consistent with the literature that monetary policy affects the housing markets through interest rate channels. On this basis, the interest rate affects the housing market through the user cost of housing, housing supply, and expectations of future housing prices movements; thereby, an increase in the user cost leads to a decrease in demand for housing, which in turn leads to a fall in housing market prices. Furthermore, an increase in interest rates may have a significant influence on housing construction costs, causing a decline in housing output. This finding corresponds with the findings of Sari (2014) who used the generalized variance decomposition approach and tested the relation between the interest rate and Turkey’s real estate market over the period from 1961 to 2000, and found findings shocks of the interest rate have noticeable effects on the housing market.

Besides, the results showed that the U.S. interest-rate coefficient is negatively and statistically significant in both the short and long-run. Thus, any decline in the U.S. interest rates leads to an increase in Turkey’s real estate market. These results confirm that the financial integration between the U.S. and Turkish markets. These results also correspond with the findings of [38] and [9] that confirmed the U.S interest rates affect the Turkish markets. Also, the results showed that the *ECT* is negative and statistically significant at a 5% level. Thus, this result confirms the long-term association among RE, DI, and USI variables. It also indicated that the fluctuations of series from the short- to long-run are amended back (4.4%) every month.

The calculated t-statistics of the lagged value of the ECT indicates that there is a long-run causality from the oil price, domestic interest rates, and the U.S. interest rates to Turkey’s real estate market (OP, DI, USI→RE). The tabulated (F)statistics values (Table 8) indicate that there is a bidirectional causality from Turkey’s real estate market to domestic interest rates (RE→DI) and from domestic interest rates to the real estate market in Turkey (DI→RE), and from oil prices to the real estate market in Turkey (OP→RE). Besides, there is a unidirectional causal relation from the U.S. interest rates to Turkey’s real estate market (US→RE), and there is a unidirectional causal relation from the U.S. interest rates to oil prices and domestic interest rate. Thus, this result confirms that there is a spillover influence of the U.S. interest rate channel on Turkey’s real estate market through oil prices and domestic interest rate factors. Therefore, this article provides evidence that the U.S. interest rates have a powerful effect on Turkey’s real estate market through oil prices, domestic interest rate channels. These empirical findings can be attributed to the presence of economic interdependence between the USA and Turkey, and the majority of the external debts and reserve currency in Turkey are composed in the USD.

**Table 8:** Results of Granger causality test.

Short-Run					Long-Run
F-stat					t-stat
(Y/X)	*ΔlnRE*	*ΔlnOP*	*ΔlnDI*	*ΔlnUSI*	ECTt−1
*ΔlnRE*	-	7.421310**	6.351230**	6.209431**	-0.054(-2.816) ***
*ΔlnOP*	3.550324	-	3.112531	6.433004**	-0.012(-1.3050)
*ΔlnDI*	6.312114**	7.62136**	-	6.452123**	-0.013(-1.1010)
*ΔlnUSI*	3.536787	3.590073	3.590073	-	-0.005 (-0.125)

** denote significance at 5% level.

## Conclusion

The research aims to provide fresh empirical evidence by testing the impact of the external shocks namely: oil prices and the U.S interest rate on Turkey’s real estate market by using three techniques of co-integration tests. The article covers the period from August 2009 to August 2018. To achieve the main objective of this research: Firstly, the article used the ADF test and the Clemente, Montanes, and Reyes (CM) test with two (SBD) to determine the order of integration of the tested variables. Secondly, the newly developed bootstrap autoregressive distributed lag (ARDL) testing model as proposed by (McNown et al. 2018), the new approach involving the Bayer-Hanck (2013) combined co-integration tests, Hatemi-J (2008) integration testing approach with (SBD) are used to provide strong evidence that the co-integration exists between the tested variables. Thirdly, the Autoregressive distributed lag testing approach (ARDL) is utilized to explore the coefficients between the variables. Finally, The Granger causality (GC) analysis is used to investigate the direction of causality among the variables. The empirical findings from the ARDL testing model indicated that oil prices have a positive influence on Turkey’s real estate market in the short and long term. Besides, the findings from the GC test demonstrate that is a unidirectional causal relation from oil prices to the domestic interest rate. This result confirms that there is a powerful effect of oil prices on Turkey’s real estate market through the domestic interest rate. However, Turkey heavily depends on imported oil; more than 50% of the energy requirement has been supplied by import. Hence, the oil price fluctuations have severe effects on economic performance in Turkeys, which in turn leads to affect the real estate market. The study suggests that the rate of imported oil in Turkey must be decreased by finding more renewable energy sources for the energy supply formula to avoid any undesirable effects of oil price fluctuations on the real estate market and also to achieve sustainable development.

Furthermore, the results of this article from the ARDL model demonstrated that there is a significant spillover influence of the U.S. interest rates on Turkey’s real estate market. Besides, the results from the GC test shows that there is a unidirectional causal relation from the U.S. interest rates to Turkey’s real estate market, and there is a unidirectional causal relation from the U.S. interest rates to oil prices and domestic interest rate. Thus, this result confirms that there is a spillover influence of the U.S. interest rate channel on Turkey’s real estate market through oil prices and domestic interest rate factors.

This study suggests the U.S interest rates may affect capital outflows, international trade, oil prices, and economic conditions in emerging economies like Turkey. Besides, the presence of economic interdependence between the USA and Turkey, and the majority of the external debts and the reserve currency in Turkey are composed in the USD, and Turkey’s oil imports hit record high in last years. All these indicators and factors led to an increase in the sensitivity and volatility of Turkey’s real estate market to oil prices and the U.S. interest rate fluctuations. Finally, this article suggests that policymakers in Turkey should pay close attention to the effects of external shocks namely the oil prices and U.S. interest rates on Turkish markets to maintain economic and financial stability.
